# Frequency of chromosomal aneuploidy in high quality embryos from young couples using preimplantation genetic screening

**Published:** 2017-05

**Authors:** Farzaneh Fesahat, Fatemeh Montazeri, Mohammad Hasan Sheikhha, Hojjatollah Saeedi, Razieh Dehghani Firouzabadi, Seyed Mehdi Kalantar

**Affiliations:** 1 *Genetics Department, Shahid Sadoughi University of Medical Sciences, Yazd, Iran.*; 2 *Research and Clinical Center for Infertility, Yazd Reproductive Sciences Institute, Shahid Sadoughi University of Medical Sciences, Yazd, Iran.*; 3 *Embryology Department, Omid Fertility Clinic, Tehran, Iran.*

**Keywords:** Preimplantation genetic screening, Aneuploidy, Sex chromosome, Autosomal chromosome

## Abstract

**Background:**

Selection of the best embryo for transfer is very important in assisted reproductive technology (ART). Using morphological assessment for this selection demonstrated that the correlation between embryo morphology and implantation potential is relatively weak. On the other hand, aneuploidy is a key genetic factor that can influence human reproductive success in ART.

**Objective:**

The aim of this lab trial study was to evaluate the incidence of aneuploidies in five chromosomes in the morphologically high-quality embryos from young patients undergoing ART for sex selection.

**Materials and Methods:**

A total of 97 high quality embryos from 23 women at the age of 37or younger years that had previously undergone preimplantation genetic screening for sex selection were included in this study. After washing, the slides of blastomeres from embryos of patients were reanalyzed by fluorescence in-situ hybridization for chromosomes 13, 18 and 21.

**Results:**

There was a significant rate of aneuploidy determination in the embryos using preimplantation genetic screening for both sex and three evaluated autosomal chromosomes compared to preimplantation genetic screening for only sex chromosomes (62.9% vs. 24.7%, p=0.000). The most frequent detected chromosomal aneuploidy was trisomy or monosomy of chromosome 13.

**Conclusion:**

There is considerable numbers of chromosomal abnormalities in embryos generated in vitro which cause in vitro fertilization failure and it seems that morphological characterization of embryos is not a suitable method for choosing the embryos without these abnormalities.

## Introduction

Aneuploidy as a key genetic factor can influence human reproductive ability. The preponderance of embryos produced by women over 37 years of age exhibit chromosomal abnormality before implantation causing developmental arrest or implantation failure (1-3). The assessment of the chromosomal constitution of in vitro generated embryos has demonstrated that chromosomal abnormalities are very frequent, with some arising from meiotic errors and others during fertilization and the first mitotic divisions (4, 5). Reduced competence of the oocyte related to maternally derived gene transcripts and stored proteins from advanced age patients may lead to chromosomal segregation errors during meiosis and/or the first embryo cleavages, with increasing risk of mosaicism (6, 7). 

However, the detection of high rates of numerical chromosomal abnormalities in embryos from young women suggests that these abnormalities are not exclusively attributed to high maternal age (8). Fluorescence in-situ hybridization (FISH) as an applicable method for selecting human oocytes and embryos by determining target euploid chromosomes offers several benefits, such as full validation and the definition of accuracy rates, besides a substantially lower cost compared with other innovations (9). Although most studies have reported valuable data about aneuploidy by using FISH this method can only permits a partial chromosomal assessment (10, 11). Therefore, it is very likely that some of the embryos classified ‘euploid’ were in fact “aneuploid” for chromosomes that were not tested, weakening the ability to detect associations between chromosomal anomalies and morphology (12-14). 

Morphological assessment for embryo selection is based on three distinct developmental stages: just after fertilization, at the cleavage stage and at the blastocyst stage (in some cases for extended embryo culture). This kind of assessment is still widely used at in vitro fertilization (IVF) clinics around the world. There are important morphological criteria at different developmental stages such as; the number of pronuclei and polar bodies, cell number, evenness of mitotic divisions, extent of blastocoel expansion and quality of inner cells mass and trophectoderm (4, 15, 16). However, the selection of the best embryo for transfer using morphological assessment provides limited information as, it is demonstrated that the correlation between embryo morphology and implantation potential is relatively weak (17, 18). Also, the relationship between chromosomal abnormality and morphological grade is still not clear. For example, top quality embryos and blastocysts were sometimes genetically abnormal with the incidence of abnormalities as high as 50%, while poor grade embryos were characterized normal (12, 13, 19). Indeed, this confirms the limited prediction power of embryo morphology on implantation rate (20).

In addition, sex selection is an attempt to control the sex of the offspring in order to achieve family balancing. It can be accomplished in several ways, in both pre- and post-implantation stages of an embryo. Two major types of pre-implantation methods can be used for social sex selection: The Ericsson method and IVF/Preimplantation genetic diagnosis (PGD) technique based on preimplantation genetic screening of sex chromosomes (21,22). The present study used FISH method to reanalyze the blastomeres for three autosomal chromosomes; 13, 18 and 21 from embryos that were selected for gender selection in couples seeking a pregnancy for a specific gender and their IVF results were not successful. 

The aim of this study was to evaluate the incidence of aneuploidy in the high quality embryos from young patients and to compare the aneuploidy rate with the pervious sex chromosomal results. In other words, this study was performed to answer the question that; if sex chromosome analysis in good morphology embryos by itself could be a valid predictor for improvement in the assisted reproductive technology (ART) outcome?

## Materials and methods

**Patients and IVF procedure**

In this lab trial study, total 23 women from 23 women at the age of 37or younger years performing IVF cycle in combination with preimplantation genetic screening (PGS) for chromosomes X and Y at the ART Unit of Omid Fertility Clinic in Tehran, Iran whom their implantations were failed, were retrospectively selected to enter this study. 

All couples had undergone intracytoplasmic sperm injection (ICSI) procedure in combination with PGS for X and Y sex selection for family balancing. A part from two, all couples already had children; they were looking for another child with a preference for one gender. Despite having had previous pregnancies, 12 couples had secondary infertility due to polycystic ovaries (n=2), male factor infertility (n=5), or both female and male factor infertility (n=5). Altogether, there were 9 couples who were fertile with a mean of 1.9 children. The inclusion criteria for patient selection were; age less than 37 years and clinical pregnancy failure at PGS/ICSI cycle.

**ICSI**

Ovarian stimulation was performed using the Gonadotropin-releasing hormone antagonist protocols including daily doses of Cetrorelix 0.25 mg (Cetrotide, Merck Serono,Germany) initiated on day 6 of stimulation or once the dominant follicle reached 14 mm and/or serum estradiol levels raised above 400 pg/ml. Recombinant human chorionic gonadotropin was administered (Ovitrelle, Merck Serono,Germany) when 2-3 follicles reached the size of ≥18 mm. Insemination was performed by ICSI. After fertilization assessment, regularly fertilized oocytes were cultured and scored at regular time intervals. The embryos were graded into four categories according to Hill: grade A; equal size blastomeres and less than 10% fragmentation, grade B; slightly unequal blastomeres with up to 20% fragmentation, grade C; unequal sized blastomeres and up to 50% fragmentation and large granules, and grade D; unequal blastomeres with significant fragmentation (>50%) and large granules (23). 

Only embryos of at least seven blastomeres with grade A and B on day 3 were selected for biopsy. At day 3 post-ICSI, embryos were placed in each microdrop of 5 µL of Ca^2+^/Mg^2+^ free biopsy medium (LG PGD biopsy medium, Life Global) under mineral oil. After zona pellucida drilling, which was performed mechanically, one nucleated blastomere was gently aspirated by a biopsy micropipette for PGS analysis. The biopsied embryo was washed twice in global total medium (Life Global) and transferred into a fresh drop of medium for further culture until the time of transfer. Each biopsied blastomere was prepared for FISH analysis of chromosomes X and Y. When the FISH analysis was completed, the corresponding slides were stored at -20°C. Embryos were selected for transfer based on FISH results of chromosomes X and Y. In addition, transfer outcome was defined by pregnant and nonpregnant patients and couples with canceled cycles because of absence the normal target gender embryos. 

**FISH procedure**

A two-round FISH procedure was performed, which allowed for the detection of chromosomes X and Y in the first round. The slides belonging to the embryos that had not led to clinical pregnancy were included in the second round and were reanalyzed for chromosomes 13, 18 and 21. Information about enumeration probe used in this study included [Xp11.1- q11.1 (DXZ1); Yp11.1-q11.1 (DYZ3) and 13q14.2; 21q22.13; 18p11.1-q11.1 (D18Z1)]. These chromosomes were selected because they are the ones most frequently involved in aneusomic pregnancies.

The FISH procedure was carried out according to the instructions with slight modifications (Cytocell, OGT Company, Cambridge, UK). In the second round, after removing the coverslip, slides were washed in PBS and dehydrated in increasing ethanol series and hybridized with the second sets of probes. Following hybridization, the slides were counterstained in 4',6-diamidino-2-phenylindole anti fade solution. Two technicians, who arrived at consensus on the diagnosis using a fluorescence microscopy (Olympus BX51 and Genetics GSL-10 with Olympus BX61; Tokyo, Japan), evaluated the results of the first and second round FISH. Microscope was equipped with the following filter sets: triple-band filters (aqua, orange, green) and single-band pass filters (red, green, aqua). Images were captured at ×60 or ×100 magnification using Specteral Imaing software. 

According to previously described scoring criteria for FISH signals, euploidy, haploidy, and polysomy were defined by the presence of two sets, one set and three or more sets, respectively, for the tested chromosomes (20). All reanalysis tests were performed by one experienced technician and confirmed by technician that carried out first sex chromosome analysis from another center, who was blind to the outcome of the embryos. The X-chromosome specific signal appeared as green and Y as red in first round. After washing, second probe set was used with fluorescent colors of green, red and blue for 13, 21 and 18 respectively.

**Ethical consideration**

The ethics committee of the Research and Clinical Center for Infertility, Yazd, Iran, approved this study. Written informed consent were obtained from all participants 

**Statistical analysis**

The data were categorical and presented in frequencies and percentages. Chi-Square test was used for statistical analysis when more than 25% of table cells have a frequency less than 5%. In tables which data frequency has a smaller amount, Fisher exact test with less power was used. Comparisons of frequency between groups, such as fertilization and developmental rates, were performed using Chi square tests by the SPSS software (Statistical Package for the Social Sciences version 20.0, SPSS Inc., Chicago, IL, USA). p<0.05 was considered as statistical significance. 

## Results

A total of 97 high quality embryos from 23 female with mean age 32.33±3.55 years were examined by FISH analysis for chromosomes X and Y (Table I). Embryo morphology assessments were performed by visual microscopic analysis that determined 82 embryos as grade A and 15 embryos as grade B. The pregnancy outcome was known for all couples, as ART cycles for five patients were canceled due to absence of euploid embryo with specific gender and the rest of them with negative clinical pregnancy after follow up. Of all the embryo transfer, 10 were females and 19 were males, with 9 single and 9 two embryos transfer. 

To investigate the chromosomal status of all 97 embryos from selected patients, rehybridization was performed with the probes specific for chromosomes 13, 18, and 21. Of 97 embryos, 36 were euploide and 61 were aneuploide (62.9%) due to monosomy, trisomy and complex abnormalities (Table II). There were 8 embryos with chromosome Y aneuploidy (7 disomy and 1 polysomy) out of 47 male embryos. 

Also, there were 9 chromosome X aneuploidy presented in 16 from 97 embryos included nine monosomy, one trisomy and three polysomy in female embryos and one disomic and two null condition in male embryos. The most frequency of autosomal chromosomes aneuploidies belonged to monosomy and trisomy (Figure I) whereas Turner (monosomy X) and Klinefelter (disomy Y) syndromes were remarkable sex chromosomes aneuploidy.

**Table I:** Patient and embryo characteristics that were selected for sex chromosome screening

**Characteristic**	**Value**
Couple	23
Primary infertility	2
Secondary infertility	12
Fertile	9
Age (years)*	32.33±3.55
Total embryo	97
Grade A	82
Grade B	15
Male embryo	47
Female embryo	50

* mean ± SD; other values were presents as thenumber.

**Table II:** Aneuploidy prevalence by chromosome

**Chromosome**	**Aneuploidy ** **/total embryos (%)**
XY	24/97(24.7)*
13	32/97(33)
18	28/97(28.9)
21	24/97(24.7)
Total	61/97(62.9)*

* There was significant difference between aneuploidy determination of embryos using sex chromosome PGS compared with combination of sex and autosomal chromosomes PGS. (62.9% vs. 24.7%, p=0.000)

**Figure I:**
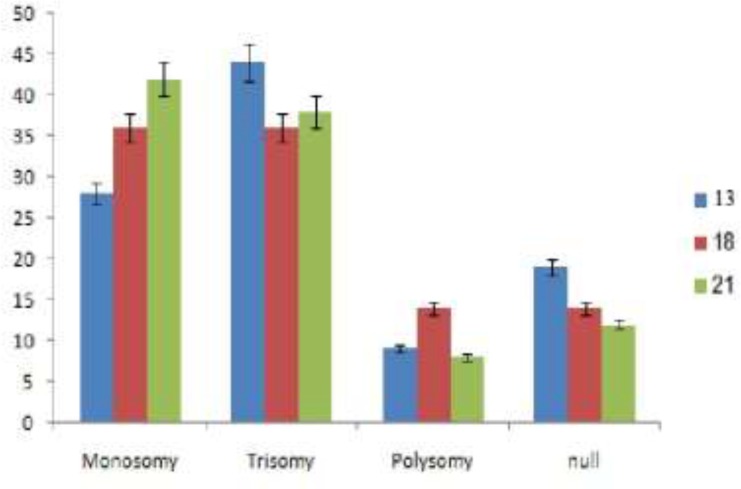
Aneuploidy type of autosomal screening chromosomes using FISH technique.

Note: Data are presented as percentage. Anueploidy type of three autosomal chromosmoses in the second round of FISH analysis.

## Discussion

In this lab trial study, all women included were relatively young (mean age 32.33±3.55 yr) and this included all the patients undergoing IVF treatment and pre-implantation genetic screening for sex selection. 

Nowadays, as new approaches are currently being developed for PGS beside IVF treatment, clinicians will more often be confronted with parental requests for transfer of an embryo of a specific sex. However, there has not been social consensus on sex selection for nonmedical reason by the European Society of Human Reproduction and Embryology (24). However, it has been tried to perform sex selection only for medical cases or for fertile couples who have at least one child and the desire of having a family with both sexes. In this study, most of the couples had one to three children and they intended to perform ICSI cycles because of secondary infertility or specific gender preferences except two couples that had no child as a result of previous abortions.

In the current study, we analyzed blastomeres from 97 embryos of 23 women for the chromosomes 13, 18 and, 21 whose abnormalities are capable of reaching term. All the embryos in our study were selected from women with negative clinical pregnancies. The transferred embryos were diagnosed as euploid according to FISH analysis of sex chromosomes. Since transfer outcomes showed different manner with negative pregnancy results, it was assumed that there was false-negative rate of the diagnosed euploid embryos and interestingly the reanalysis results of five chromosomes verified the hypothesis with significantly higher frequency of aneuploidies than first sex FISH results (p<0.001) (Table II).

Kilani *et al* in 2014 performed a study with the aim of investigating correlation with embryo viability and the level of FISH sensitivity (embryos confirmed to be euploid) on 173 young couples of proven fertility who had previously undergone preimplantation genetic screening for chromosomes X and Y for family balancing. They concluded that the proportion of euploid embryos was significantly lower in 53 non-pregnant women when compared with 99 women with term pregnancy (49% versus 75% respectively, p<0.001). Moreover, all embryos transferred were found to be chromosomally abnormal in 21 non-pregnant patients after reanalyzing using PGS (25). These results were in agreement to our findings. 

Some evidence suggests that about 60% to 90% of all transferred embryos in IVF cycles do not implant and also, embryonic factors may be the basic reasons for large percentage of implantation failures (26). Indeed, recent investigations indicate that the embryo selection should not be based only on morphological assessment and modern methods must be used (27, 28). Our results confirmed that morphological embryo observation is inadequate for selections of normal embryos as all embryos entered the present study were categorized morphologically in top grades.

In another study, Eaton and colleagues examined the relationship between embryo morphology on cleavage stage embryos and aneuploidy of some chromosomes. They observed that the embryos with high quality were more likely to be euploid for certain chromosomes, including X/Y, 8, 15, 16, 18, and 22, whereas other chromosomes such as 13, 20, or 21 did not show any obvious effect on either cleavage stage morphology or blastocyst development (11). In this study, the most frequent chromosome aneuploidy belonged to chromosome 13 and not sex chromosomes. Of course, sample size of these studies may be an enough reason for some differences between our observations comparing with them. However, as it is shown in table II, it is clear that aneuploidy were more common in autosomal chromosomes (13 and 18) than sex chromosome.

Recently, comprehensive chromosomal screening for all chromosomes is suggested using single nucleotide polymorphism array, comparative genomic hybridization array or quantitative PCR which is more informative compared to the FISH techniques evaluated fewer than half of the chromosomes in each embryo (12-14, 19). However, it was shown that the five-chromosome test detects 28-31%, nine probes detect 70-72% and the 12-probe test detects 79-80% of chromosome abnormalities found in fetuses (9). Also, some legal and technical restrictions as well as cost-related issues cause applying advanced techniques may not be possible for the number of couples (2, 29).

Several new studies described the significant improvement in reproductive outcomes with the use of morphokinetic model using time lapse imaging to identify embryos at risk for aneuploid chromosomes (30, 31). Campbell by using both single nucleotide polymorphic and comparative genomic hybridization arrays from trophectoderm biopsies and time laps imaging showed aneuploidy rate of 60% which was related to delayed initiation of blastocyst formation in anueploid embryos compared to euploid embryos (32, 33). 

There is considerable numbers of in vitro generated embryos tends to be chromosomally abnormal while morphological characterization of embryos doesn't completely consistent with chromosomal content. Also, chromosomal abnormalities of embryos are one of the most critical reasons for poor ICSI outcomes. Therefore, it is suggested that new non-invasive techniques applies for embryo selection such as time lapse imaging for dynamic morphokinetic analysis and cytogenetic assessments of at least prevalent autosomal chromosomes besides sex chromosomes.

## Conclusion

It seems that the ART improvement is directly related to the number and types of chromosomes analyzed. Complete karyotypes obtained from early embryos using CGH and microarray analyses is the most comprehensive test available for detecting chromosomal abnormalities while when these advanced techniques are not available it is shown that even by using only five probes (X,Y, 13, 18, 21) a reduction in spontaneous abortions has been achieved. 

## Note

***This article extracted from the Ph.D. thesis. (Farzaneh Fesahat)***
